# Effect of Probiotics/Prebiotics on Cattle Health and Productivity

**DOI:** 10.1264/jsme2.ME14176

**Published:** 2015-05-23

**Authors:** Yutaka Uyeno, Suguru Shigemori, Takeshi Shimosato

**Affiliations:** 1 Faculty of Agriculture, Shinshu UniversityMinamiminowa, Nagano 399–4598Japan; 2 Interdisciplinary Graduate School of Science and Technology, Shinshu University8304 Minamiminowa, Kamiina, Nagano 399–4598Japan; 3 Research Fellow of the Japan Society for the Promotion of Science (JSPS)

**Keywords:** rumen, gastrointestinal tract, yeast, oligosaccharide

## Abstract

Probiotics/prebiotics have the ability to modulate the balance and activities of the gastrointestinal (GI) microbiota, and are, thus, considered beneficial to the host animal and have been used as functional foods. Numerous factors, such as dietary and management constraints, have been shown to markedly affect the structure and activities of gut microbial communities in livestock animals. Previous studies reported the potential of probiotics and prebiotics in animal nutrition; however, their efficacies often vary and are inconsistent, possibly, in part, because the dynamics of the GI community have not been taken into consideration. Under stressed conditions, direct-fed microbials may be used to reduce the risk or severity of scours caused by disruption of the normal intestinal environment. The observable benefits of prebiotics may also be minimal in generally healthy calves, in which the microbial community is relatively stable. However, probiotic yeast strains have been administered with the aim of improving rumen fermentation efficiency by modulating microbial fermentation pathways. This review mainly focused on the benefits of probiotics/prebiotics on the GI microbial ecosystem in ruminants, which is deeply involved in nutrition and health for the animal.

## Overview

The gastrointestinal (GI) microbial community, which consists of at least one thousand different microbial species in human gut (14, 84), has an impact on energy efficiency in the host, including energy intake, transport, conversion, and storage. In ruminants, a large amount of energy recovery from dietary polysaccharides that cannot be digested by the host has been attributed to the function of the microbial community in the rumen; however, this process also depends on the structure of the microbiota inhabiting this organ. Environmental and stochastic factors, such as diet composition, feeding practices, and farm management, have been shown to strongly affect the composition and functions of the microbiota in livestock animals (83).

Most of the GI bacterial community of mammals is affiliated with two phyla, *Bacteroidetes* and *Firmicutes* (1, 39, 77). On the other hand, other phyla have niches in each community, depending on the animal species. Therefore, the GI tract community is unique among species, which require owning different systems to efficiently convert their diet into their energy. The main GI bacterial groups in cattle have been identified as defined groups (mainly genera) for up to 90% of the total community (78, 79). However, a certain proportion of the gut bacterial community has yet to be identified due to an incomplete understanding of the bacterial community structure in GI ecosystems because many of the 16S rRNA gene sequences recovered from fecal samples are derived from unknown (*i.e.*, not previously identified in the intestinal microbiota) species (21). While GI tract ecosystems (especially in humans) are known that a higher proportion of bacteria has been cultivated (18), further research is required to uncover a larger proportion of the unknown microorganisms abundantly present in the GI microbiota.

The term “probiotics” has been amended by the FAO/ WHO to “Live microorganisms, which, when administered in adequate amounts, confer a health benefit on the host” (24). Several lactic acid bacteria (LAB) strains, species belonging to the genera *Lactobacillus*, *Bifidobacterium*, and *Enterococcus*, are considered beneficial to the host and have, thus, been used as probiotics and included in several functional foods. Probiotics have the ability to enhance intestinal health by stimulating the development of a healthy microbiota (predominated by beneficial bacteria), preventing enteric pathogens from colonizing the intestine, increasing digestive capacity, lowering the pH, and improving mucosal immunity. It is important for the introduced microbes not to disturb the indigenous population, which has already been adapted to the environment of the GI tract to work both for and with the host. Additionally, there are a number of requirements for allochthonous probiotic strains to adapt to the intestinal environment of an animal species, *e.g.*, bile acid tolerance and affinity to the intestinal mucosa and glycoproteins. The situation in the rumen is similar; ingested microbes have to find out a suitable niche to inhabit, such as the rumen epithelium, rumen fluid, or fibrous feed, and exert effects on the health of the host, such as the removal of toxic molecules and digestion of polymeric carbohydrates.

Prebiotics are non-digestible food ingredients that, when consumed in sufficient amounts, selectively stimulate the growth and/or activity of one or a limited number of microbes in the gut. Impacts of orally administered probiotics (in this case, referred to as symbiotics) and intrinsic beneficial bacteria of the GI tract can be enhanced by the use of prebiotics (28). The most commonly used prebiotics to yield health benefits are carbohydrate substrates, such as oligosaccharides or dietary fiber with low digestibility.

Research on probiotics and prebiotics has developed as a collaborative study domain between the fields of food and feed with medicine and pharmaceutics. There are also a number of application studies for cattle; however, few have been discussed in association with the dynamics of the inherent microorganisms. This review explored the better usage of probiotics and prebiotics to improve ruminant performance by discussing the possible impacts of the applications of probiotics and prebiotics on the ruminant-specific GI microbial community.

## Gastrointestinal microbial structure of cattle

Neonatal ruminants are unique in that, at birth, they are physically and functionally two different types of animal with respect to their GI system (34). The intestine of a newly born calf is sterile, and colonization of the GI tract begins immediately after birth. Thereafter, a complex and dynamic microbial ecosystem with high densities of living bacteria is established in the large intestine as animals grow to maturity (72). Molecular-based monitoring of the intestinal bacterial communities of calves revealed that the community undergoes dynamic changes during the first 12 weeks of life (78). For example, the main groups detected at a very young age (less than 3 weeks old) were found as major populations in the human fecal bacterial community (*i.e.*, *Bacteroides*-*Prevotella*, the *Clostridium coccoides*-*Eubacterium rectale* group, *Faecalibacterium*, and *Atopobium*) (30, 70, 77). *Bacteroides-Prevotella* and the *C. coccoides-E. rectale* group comprised a major fraction of the microbiota (*ca.* 50%–70% of the total) throughout the first 12-week period after birth, whereas the numbers of *Atopobium*, *Faecalibacterium*, and some probiotic bacteria (such as those of the genera *Lactobacillus* and *Bifidobacterium*) decreased as the animal aged. Instead, an uncultivated rumen bacterial group as well as *Ruminococcus flavefaciens* and *Fibrobacter* emerged at detectable levels (1%–2%) in feces sampled after weaning. Changes in the GI microbiota of young calves are in accordance with the metabolic and physiological development of the GI tract (15). As discussed later, this immature and fluctuating gut microbiota has to face an abrupt change in diet, which leads to an increase in the susceptibility of young animals to pathogen colonization and subsequent diarrhea and respiratory disease.

GI microbial communities are involved in the digestion and fermentation of plant polymers, which is of particular importance in mature herbivorous animals. Ruminant animals harbor a complex microbial community consisting of a diverse array of anaerobic microbes in the rumen, which forms a different community structure from aerobic consortia for fiber digestion (20). These microorganisms interact with one another and take part in the systematic digestion of fibrous plant material, which they anaerobically ferment into end products that are, in turn, used as energy sources by the host (66). Microbial characteristics, functions, and current concerns regarding dysfunctions in the two respective sites (*i.e.*, the rumen and lower intestine) in cattle are summarized in Table 1. Even though the major functionalities differ from each other, ruminal community may affect that of large intestine.

Numerous factors, such as dietary and management constraints, can strongly affect the structure and activities of these microbial communities, sometimes leading to impaired health and performance in livestock animals (16). For example, sub-acute ruminal acidosis (SARA) is a well-recognized digestive dysfunction that is increasingly becoming a health problem. Microbial community changes associated with SARA of lactating dairy cattle have been monitored using terminal-restriction fragment length polymorphisms (T-RFLP) of 16S rRNA genes and real-time PCR (40). Different rumen microbial population structures between grain- and forage-induced SARA were observed even though rumen fermentation conditions were similar. The findings of a T-RFLP analysis indicated that the most predominant shift during SARA was a decline in Gram-negative *Bacteroidetes*. Since inflammatory responses may be attributed, in part, to lipopolysaccharides released by the dead cells of Gram-negative bacteria (44, 58), the change observed in the number of *Bacteroidetes* in response to SARA appears to be reasonable. The potential microbial and physiological factors that increase the incidence of SARA by enhancing the epithelial permeability of lipopolysaccharides have not yet been identified. Furthermore, the overconditioning (excess body fat deposition) of dairy cows is a major risk factor for metabolic, infectious, digestive, and reproductive disorders (68). However, the efficient fermentation system in the rumen of dairy cows is primarily responsible for the conversion and accumulation of energy, while the colonial microbiota is majorly contributed to energy harvesting and metabolism in human (14, 38, 45). This implies the importance of controlling rumen microbial fermentation, although rumen function is not directly related to body fat deposition.

## Current applications of probiotics in calves

In young pre-ruminants, probiotics such as LAB or *Bacillus* species generally target the lower intestine and represent an interesting means to stabilize the gut microbiota and decrease the risk of pathogen colonization. LAB are well-known probiotic supplement for young calves, and are regarded as applicable to regular feeding practices. Previous findings support the beneficial effects of these products in balancing the GI tract microbiota as well as in animal nutrition and health (Table 2). Diarrhea is the main cause of morbidity and mortality in calves during their early life (13, 36); therefore, its prevention is important to promote the growth of calves (69, 76). Antibiotic therapy has been applied to maintain the performance of calves and reduce scours. However, due to increasing safety concerns regarding the risks of antibiotic resistance due to the release of antibiotics into the environment and persistence of chemical residues in animal products (49, 82), probiotic additives have been developed as alternatives to improve animal health and productivity (4, 8). Although the administration of probiotics to animals has been linked with efficacy on specific groups (pathogens) in the gut microbiota (25, 69, 76), how they interact with the whole gut community currently remains unclear. As discussed above, lactobacilli and bifidobacteria numbers have been shown to decrease in the community in the early stages of life in cattle (78). Optimizing the enteric flora is considered effective for healthy calf rearing because it increases the numbers of such beneficial microorganisms. The supply of microorganisms together with feed from birth in a preventive manner allows the incorporation and establishment of these probiotic strains together with the microbiota of calves. In addition, early colonization by LAB in the intestinal ecosystem may decrease the adherence of pathogens to the intestinal mucosa (37). A stable microbial load of *Lactobacillus* species has been shown to improve weight gain and immunocompetence in young calves (3); however, previous findings regarding the use of probiotics in calf feeding have generally been equivocal, as shown in Table 2. The efficacy of probiotic strains may vary depending on whether calves are raised under healthy conditions because, in previous studies, the effects of probiotics were often significant when control (untreated) calves were less healthy, as determined from fecal scores or rectal temperatures (5, 76). Under stressed conditions, direct-fed microbials may be used to reduce the risk or severity of scours caused by disruption of the normal intestinal environment. A better understanding of how the selected lactobacilli and bifidobacteria strains overcome the effects of pathogens, by antagonizing the pathogenicity, and/or modulating the immune responses to infections is needed (3, 69).

## Current applications of prebiotics in calves

Several types of oligosaccharides have been suggested to have specific functionalities in calves. Mannan oligosaccharides (MOS) are complex mannose sugars that are believed to block colonization of pathogens in the digestive tract. A previous study demonstrated that deeding fructooligosaccharides (FOS) in combination with spray-dried bovine serum to calves reduced the incidence and severity of enteric disease (62). It has been suggested that this sugar prevents the adhesion of *Enterobacteriaceae*, most notably *Escherichia coli* and *Salmonella*, to the intestinal epithelium (7, 31). Galactosyl-lactose (GL) is a trisaccharide (galactose plus lactose) that is produced by the enzymatic treatment of whey with beta-galactosidase. The addition of GL to milk replacer (MR) was previously found to have beneficial effects on the growth and health of dairy calves (61). Supplementation with MOS, FOS, and GL may improve the growth performance of calves in either the pre- or postweaning stage; however, modifications to the activities of microbial fermentation by these sugars have not yet been examined in detail. In addition, similar to the case of probiotics, the observable benefits of prebiotics are likely to be minimal when calves are generally healthy (35). As shown in Table 2, most prebiotics may not have any apparent beneficial effects (body weight gain, feed efficiency, or health measures) over probiotics.

We previously evaluated the effects of feeding cellooligosaccharide (CE), which is a commercially available oligosaccharide that consists of glucose with beta-1-4 linkages, on performance and intestinal ecology in Holstein calves fed MR or whole milk (80). No significant differences were observed in fecal bacterial community compositions or organic acid profiles in the MR group. However, this supplementation appeared to effectively modulate the intestinal bacterial community of calves when administered with whole milk because the proportion of the *C. coccoides*–*E. rectale* group was higher in the prebiotic group in the whole milk-feeding trial. From these results, type of liquid feed (MR or whole milk) to preweaned calves may be responsible for the different responces to feeding CE. Overall, CE supplementation had no effect on the maintenance of *Lactobacillus* and *Bifidobacterium* species levels in the large intestine of preweaning calves. CE is considered to be utilized by specific microbes inhabiting the calf intestine, resulting in increases in the number of butyric acid-producing bacteria belonging to *C. coccoides*–*E. rectale* (19, 47). Fecal butyrate concentrations were also higher at that time. Along with its value as an energy source, butyrate is also involved in the growth and differentiation of intestinal cells in the large intestine, thereby improving its epithelial structure (59) and enhancing digestion and absorption efficiencies, which may also contribute to a superior ability for nutrition capture (54). CE fed with liquid feed (milk or reconstituted MR) may reach the lower digestive tract vis the esophageal groove reflex (34), and exert prebiotic effects using a similar mechanism to that of monogastric animals.

An *in vivo* study indicated that CE feeding improved daily gain and feed efficiency in calves during the postweaning period, but not the pre-weaning period (32). This may have been mainly due to the enhancement in ruminal fermentation as propionate and total short chain fatty acid (SCFA) levels were increased, which suggests that CE affected the fermentation pattern by providing carbon and energy sources (46). After weaning, solid feeds directly reach the rumen and are then microbially processed. Ruminal CE may eventually be a source of nutrition for various types of indigenous microbes. With the exception of very young ruminants, prebiotics orally administered to ruminants are consumed by ruminal microbes and fail to reach the lower intestine unless protected from ruminal digestion. The administration of oligosaccharides to weaned calves still appears to be advantageous because the formation of a desirable intestinal (rumen and/or lower intestine) community in calves through prebiotic supplementation may contribute to further improvements in growth performance at an older age.

## Effects of supplementation with probiotics/prebiotics on the performance of heifers, lactating cows, and beef cattle

Probiotics for adult ruminants have mainly been selected to improve fiber digestion by rumen microorganisms. Such probiotics have positive effects on various digestive processes, especially cellulolysis and the synthesis of microbial proteins. The main form of probiotic commonly used in dairy cows is various strains of yeast (mostly *Saccharomyces cerevisiae*). Regarding bacterial probiotics for adult ruminants, lactate-producing bacteria (*Enterococcus*, *Lactobacillus*), which sustain lactic acids are a more constant level than *Streptococcus bovis*, may represent a possible means of limiting acidosis in high-concentrate-fed animals (55, 56), especially feedlot cattle. *Megasphaella elsdenii* or *Propionibacterium* species, which utilize lactate, have also been administered as directfed microbials to avoid the accumulation of ruminal lactate (26, 41, 71).

The most consistent effects following the addition of yeast cultures to the diet include improved productivity in both lactating and growing animals. The mode of action of yeast products has not yet been elucidated in detail, but is generally considered to involve changes in rumen fermentation rates and patterns. Certain strains of active dry yeast are particularly effective at raising and stabilizing ruminal pH by stimulating certain populations of ciliate protozoa, which rapidly engulf starch and, thus, effectively compete with amylolytic lactate-producing bacteria (2, 9, 41, 56, 73, 75). A less acidic ruminal environment has been shown to benefit the growth and fiber-degrading activities of cellulolytic microorganisms (6, 10, 12, 52). Yeast also has the potential to alter the fermentation process in the rumen in a manner that reduces the formation of methane (CH_4_) gas (12). In a previous study, commercial yeast product slightly decreased CH_4_ in growing beef cattle, while neither the SCFA amount nor the profiles changed (51). The cells of *S. cerevisiae* provide growth factors for rumen microbes, including organic acids and oligosaccharides, B vitamins, and amino acids, which stimulate microbial growth in the rumen, thereby indirectly stabilizing ruminal pH (50). Another function of yeast in the rumen is the scavenging of oxygen, which creates the more anaerobic environment required by ruminal microorganisms (16). In this context, yeast itself functions not only as a probiotic, but also helps other rumen community members grow, and, thus, acts as a type of prebiotic. The effects of active dry yeast on the rumen microbial community structure was recently determined by 16S rRNA gene-based clustering using a pyrosequencing technique (57). An evaluation of the effects of yeast on the microbiota revealed that some bacterial groups were more affected than others. The relative abundance of lactate-utilizing bacteria such as *Megasphaera* and *Selenomonas* as well as fibrolytic groups (*Fibrobacter* and *Ruminococcus*) increased with yeast supplementation, confirming improvements in cellulolytic activity as a supposed mode of action of yeast.

Intervention studies on the application of commercial yeast cultures to young cattle (heifers and calves) are summarized in Table 2. A large number of studies that evaluated the effects of yeast on dairy production (milking and body mass deposition) were summarized in two studies published concurrently several years ago, one of which was a meta-analysis and the other was a review (17, 63). Desnoyers *et al.* evaluated the effects of yeast supplementation on intake, milk production, and rumen fermentation characteristics using a quantitative meta-analysis. The positive effects of yeast supplementation were an increase in rumen pH and a decrease in lactic acid with the increases in concentrate in the diet and with the intake level. Controversially, the positive effects of yeast supplementation on organic matter digestibility increased with the percentage of fiber in the diet, suggesting an improvement in rumen fermentation by yeast supplementation.

In beef cattle, the stabilization of ruminal pH may also be effective when they are fed a high readily fermentable diet that increases the risk of acidosis. Growth parameters (average daily gain, final weight, intake, and feed to gain ratio) were previously reported to be improved by continuous live yeast supplementation (11), whereas no or little effect on performance was observed in other studies (6, 81). This difference in result may be attributed to a primal difference in the rumen microbial composition, in which respective members have different pH tolerances. For example, fibrolytic bacteria are generally less pH tolerant than saccharolytic bacteria (65).

Although previous findings have supported the efficacy of yeast supplementation, conclusive evidence has not yet been obtained to show that supplementation is beneficial at all times (11). It should be noted that this potential varies markedly with products (51). Increases in profitability are generally variable, especially when taking the rise in feeding costs for these products into account. Some of these differences may be attributed to the type and strain of yeast used as well as whether the cells are alive or dead (48). Furthermore, in some commercial products, the data available have been generated under *in vitro* conditions and in monogastric animals or small ruminants, which do not necessarily correspond to actual dairy and beef production.

## Concluding remarks

The cattle GI microbial composition was shown to be altered by various factors, including diet, age, and stress, as an adaptive response of the community to the environment (66). Therefore, GI health may be defined as the ability to maintain a balance of GI ecosystem. Desirable community shift may be attributed to the effect of probiotics and prebiotics, rather than autonomic change. Probiotics and prebiotics both have great potential in livestock productivity as well as human health. Cellooligosaccharide is one example because many rumen bacteria may be able to use it, but, when administered to preweaned calves, the proportion of the *C. coccoides–E. rectale* group specifically increases in the lower intestine. Like CE, it may become possible to use powerful materials that work either or both on the rumen and on the lower intestine.

Although controlled studies demonstrated that probiotics and prebiotics achieved a positive balance in the GI microbiota of cattle, the dynamics and functions of the rumen community need to be examined in more detail. Further studies on the structure and activities of the gut microbiota, functional interactions between gut microbes, and relationships between microbes and host cells are warranted to determine the fundamental aspects of future probiotic/prebiotic research. “Meta-omic” approaches (metagenomic, metatranscriptomic, metaproteomic, and meta-metabolomic analyses) are powerful tools for analyzing the relationships between the GI microbial community and host metabolism (64, 67, 84). Future meta-omic-based studies together with the knowledge obtained to date will provide deeper insights into the effects of “health-improving” diet for animals by better characterizing and understanding the functionalities of probiotics on the balance of the GI microbiota.

## Figures and Tables

**Table 1:** Cattle GI microbial characteristics and relationships with host health and performance

	Rumen	Large intestine
Major groups in microflora	*Bacteroidetes* *Firmicutes* *Fibrobacter* *Archaea* Protozoan species	in preweaned calves: *Bacteroidetes* *Firmicutes* *Atopobium* *Bifidobacteria* in weaned calves or older cattle: *Bacteroidetes* *Firmicutes* (including uncultured groups) *Fibrobacter*
Major microbial functions	Involved in host nutrition (digestion of fibrous plant material and anaerobic fermentation to short chain fatty acids, which can be used as an energy source by the host; microbial protein synthesis)	Immunological responses Digestion of polymers
Microbial dysfunctions	Overgrowth of lactate-producing bacteria, leading to a decrease in rumen pH and subsequent rumen acidosis Decrease in microbial activity by unbalanced nutrition, leading a decrease in feed protein efficacy	Pathogenesis by harmful bacteria, such as *E. coli* and *Salmonella*.

**Table 2:** Recent probiotic/prebiotic trials applied for young cattle

Targets and materials applied^a^	Positive effects in respect to			Remarks	Reference

	Weight gain	Feed efficiency	Health		
*Probiotics (for heifers):*					
Yeast culture	Not assessed	Yes	No		(42)
Yeast culture	No	No	Yes		(53)
*Probiotics (for calves)*:					
Yeast culture	Yes	No	No		(43)
MSPB or CSPB	Yes	Yes	Yes	Effects were determined when the results of four experiments were pooled.	(76)
MSPB	Yes	No	Not assessed	Two mixtures were tested, a commercial probiotic and laboratory-produced probiotic that was made under laboratory conditions.	(5)
*Lactobacillus casei* ssp. *casei*	Yes	No	Yes	Synbiotic trial	(32)
MSPB^b^	Yes	No	Yes		(22)
MSPB^b^	No	No	No		(23)
*Prebiotics (for calves):*					
FOS	No	No	Yes		(62)
FOS (short chain)	No	Yes	Not assessed		(29)
MOS	No	No	Yes		(33)
MOS	No	No	No		(74)
MOS	Yes	Yes	Yes	Used crossbred calves	(27)
Cellooligosaccharide	Yes	No	Yes	Synbiotic trial	(32)
A commercial product^c^	No	No	No		(35)
A commercial product^c^	No	No	No	The lactobacilli count in feces was higher and that of bifidobacteria was slightly higher in the prebiotic group.	(60)

a MSPB, multi-species probiotic; CSPB, calve-specific probiotic; MOS, mannan-oligosaccharides.

b A mixture of *Lactobacillus casei* subsp. *casei*, *Lactobacillus salivarius*, and *Pediococcus acidilactici*.

c Derived from a cell-free culture of a *Propionibacterium freudenreichii* strain.
